# Bed‐rest and exercise remobilization: Concurrent adaptations in muscle glucose and protein metabolism

**DOI:** 10.1002/jcsm.13431

**Published:** 2024-02-12

**Authors:** Natalie F. Shur, Elizabeth J. Simpson, Hannah Crossland, Despina Constantin, Sally M. Cordon, Dumitru Constantin‐Teodosiu, Francis B. Stephens, Matthew S. Brook, Philip J. Atherton, Kenneth Smith, Daniel J. Wilkinson, Olivier E. Mougin, Christopher Bradley, Ian A. Macdonald, Paul L. Greenhaff

**Affiliations:** ^1^ Centre for Sport, Exercise and Osteoarthritis Research Versus Arthritis, School of Life Sciences University of Nottingham, Queen's Medical Centre Nottingham UK; ^2^ National Institute for Health and Care Research (NIHR) Nottingham Biomedical Research Centre Nottingham University Hospitals NHS Trust and University of Nottingham, Queen's Medical Centre Nottingham UK; ^3^ MRC/Versus Arthritis Centre for Musculoskeletal Ageing Research, Schools of Life Sciences and Medicine University of Nottingham, Queen's Medical Centre Nottingham UK; ^4^ Sport and Health Sciences University of Exeter Exeter UK; ^5^ Sir Peter Mansfield Imaging Centre, School of Physics University of Nottingham Nottingham UK

**Keywords:** human metabolism, immobilization, insulin sensitivity, leg glucose uptake, muscle atrophy, muscle gene expression, muscle glycogen storage, muscle protein breakdown, muscle protein synthesis, rehabilitation

## Abstract

**Background:**

Bed‐rest (BR) of only a few days duration reduces muscle protein synthesis and induces skeletal muscle atrophy and insulin resistance, but the scale and juxtaposition of these events have not been investigated concurrently in the same individuals. Moreover, the impact of short‐term exercise‐supplemented remobilization (ESR) on muscle volume, protein turnover and leg glucose uptake (LGU) in humans is unknown.

**Methods:**

Ten healthy males (24 ± 1 years, body mass index 22.7 ± 0.6 kg/m^2^) underwent 3 days of BR, followed immediately by 3 days of ESR consisting of 5 × 30 maximal voluntary single‐leg isokinetic knee extensions at 90°/s each day. An isoenergetic diet was maintained throughout the study (30% fat, 15% protein and 55% carbohydrate). Resting LGU was calculated from arterialized‐venous versus venous difference across the leg and leg blood flow during the steady‐state of a 3‐h hyperinsulinaemic–euglycaemic clamp (60 mU/m^2^/min) measured before BR, after BR and after remobilization. Glycogen content was measured in *vastus lateralis* muscle biopsy samples obtained before and after each clamp. Leg muscle volume (LMV) was measured using magnetic resonance imaging before BR, after BR and after remobilization. Cumulative myofibrillar protein fractional synthetic rate (FSR) and whole‐body muscle protein breakdown (MPB) were measured over the course of BR and remobilization using deuterium oxide and 3‐methylhistidine stable isotope tracers that were administered orally.

**Results:**

Compared with before BR, there was a 45% decline in insulin‐stimulated LGU (*P* < 0.05) after BR, which was paralleled by a reduction in insulin‐stimulated leg blood flow (*P* < 0.01) and removal of insulin‐stimulated muscle glycogen storage. These events were accompanied by a 43% reduction in myofibrillar protein FSR (*P* < 0.05) and a 2.5% decrease in LMV (*P* < 0.01) during BR, along with a 30% decline in whole‐body MPB after 2 days of BR (*P* < 0.05). Myofibrillar protein FSR and LMV were restored by 3 days of ESR (*P* < 0.01 and *P* < 0.01, respectively) but not by ambulation alone. However, insulin‐stimulated LGU and muscle glycogen storage were not restored by ESR.

**Conclusions:**

Three days of BR caused concurrent reductions in LMV, myofibrillar protein FSR, myofibrillar protein breakdown and insulin‐stimulated LGU, leg blood flow and muscle glycogen storage in healthy, young volunteers. Resistance ESR restored LMV and myofibrillar protein FSR, but LGU and muscle glycogen storage remained depressed, highlighting divergences in muscle fuel and protein metabolism. Furthermore, ambulation alone did not restore LMV and myofibrillar protein FSR in the non‐exercised contralateral limb, emphasizing the importance of exercise rehabilitation following even short‐term BR.

## Introduction

Bed‐rest (BR) of a few days duration is common during hospitalization, trauma and ill‐health. Both BR and unilateral leg immobilization (3–7 days) are accompanied by skeletal muscle atrophy, and reductions in muscle protein synthesis and insulin‐stimulated whole‐body and leg glucose disposal (GD).^1^, ^2^, ^3^, ^4^ The recovery of metabolic homeostasis during remobilization following BR and immobilization has however not been well explored, particularly under conditions where simultaneous measurements of limb glucose uptake and muscle protein synthesis have been quantified concurrently in the same volunteers in longitudinal study designs. This represents an important gap in understanding the recovery of metabolic homeostasis following episodic health burdens.

Upper limb immobilization of just 24‐h duration has been shown to reduce insulin‐stimulated forearm GD by 38%, which was specific to the immobilized limb.^5^ In keeping with this, 7 days of BR also resulted in reduced insulin‐stimulated leg glucose uptake (LGU), which was of a greater magnitude than that seen at the whole‐body level.^4^ Collectively, these observations highlight that immobilization‐induced reductions in limb insulin‐stimulated GD are rapid and most likely reside at a muscle level as a result of a lack of muscle contraction per se. It is not unreasonable, therefore, to suggest that a similarly short‐duration exercise intervention following BR could rapidly restore insulin‐stimulated limb GD to the pre‐immobilized state; however, this is currently unresolved. A single bout of exercise in non‐immobilized, healthy, young volunteers acutely increased insulin‐stimulated LGU,^6^ and this has been shown to diminish over the 48–72 h following exercise.^7^ In a study involving 21‐day BR in healthy, sedentary young males maintained in energy balance, 5–14 days, but not 4 days, of habitual free living was able to restore whole‐body glucose tolerance in response to an oral glucose challenge.^8^ Another study reported that a significantly longer period (8 weeks) of progressive eccentric resistance exercise training (knee and hip extensors three times per week) was required to restore insulin sensitivity measured using the CLIX‐IR, an index of insulin sensitivity derived from an oral glucose tolerance test (OGTT), following 5 days of BR.^9^ It is possible, however, that this duration of free living alone could have restored insulin sensitivity without exercise training intervention. It is currently unknown whether a short period (3 days) of structured resistance exercise‐supplemented remobilization (ESR) can restore any BR‐induced decrements in whole‐body GD and LGU and muscle glycogen storage.

Both BR and single‐limb immobilization result in muscle mass loss. Studies of only a few days duration, however, are sparse. One study, using dry immersion as a model of immobilization, reported a 2.4% loss of quadriceps cross‐sectional area (CSA) after 3 days.^10^ Disuse muscle atrophy is underpinned by a net imbalance between rates of muscle myofibrillar protein synthesis (MPS) and muscle protein breakdown (MPB), and hypothetically, atrophy could result from a reduction in MPS, an increase in MPB or both. Studies using stable isotopically labelled amino acid tracers have demonstrated in healthy men that decrements in rates of postabsorptive MPS can account for the majority of muscle atrophy during immobilization,^11^, ^12^ in addition to blunted MPS responses to protein feeding.^12^ Animal models have suggested that MPB is an important driver of disuse atrophy in the first few days of disuse.^13^, ^14^ However, evidence in humans is less clear, largely due to technical difficulties of directly measuring MPB *in vivo*. Tesch et al. pointed to an increase in MPB after 72 h of unilateral leg immobilization in humans, demonstrated by a 44% increase in interstitial 3‐methylhistidine within the *vastus lateralis*, measured using microdialysis.^15^ Conversely, a more recent study reported a reduction in daily cumulative MPS, but no change in MPB after 4 days of unilateral immobilization in healthy men, measured using an acute pulse‐chase tracer decay rate technique.^1^

The restoration of decrements in muscle volume and MPS following short‐duration immobilization has not been well explored. Six weeks of isokinetic resistance exercise (five bouts of 30 maximal isokinetic knee extensions, each separated by 1 min, at a velocity of 180°/s on three occasions per week) was reported to restore the 4.7% loss of leg lean mass observed following 14 days of leg immobilization.^16^ Furthermore, the restoration of lean mass at 1, 4 and 6 weeks of the exercise intervention was associated with the restoration of isometric strength (*P* < 0.01). Human volunteer research has also shown that the decline in fed‐state leg MPS after 5 days of BR was restored after 8 weeks of high‐intensity resistance exercise rehabilitation in the form of four 5‐min intervals of lower limb high‐intensity eccentric contractions, three times per week.^17^ There is currently a lack of evidence regarding the impact of short‐duration (<5 days) muscle reloading, using resistance exercise, on BR‐induced decrements in muscle volume and MPS and how these responses differ from post‐BR habitual free living alone.

Collectively, these studies identify a gap in our understanding of the metabolic regulation of post‐immobilization muscle rehabilitation. To address this gap, the present study describes novel changes in insulin‐stimulated LGU and muscle glycogen storage, alongside concurrent changes in muscle volume, chronic MPS and whole‐body MPB during 3 days of BR and three subsequent days of controlled remobilization in healthy young male volunteers. The findings highlight BR‐induced adaptations in these endpoint measures and also novel responses in muscle glucose and protein metabolism in response to resistance ESR.

## Methods

### Experimental model and subject details

Ten healthy physically active males were recruited from the general population and enrolled to undertake the study at the University of Nottingham (UK) if they met inclusion criteria (listed previously in Shur et al.^2^) and passed a medical screening. The protocol consisted of a 7‐day run‐in phase where diet was controlled and pre‐BR experimental measures were collected, followed by a 3‐day −6° head‐down tilt (HDT) BR phase and a 3‐day structured remobilization phase (*Figure* *S1a*). Experimental sessions (*Figure* *S1b*) were performed during the run‐in phase (4 days prior to BR), 3 days (72 h) after commencing BR and 3 days after structured remobilization. Further details of each phase are provided in the supporting information.

Individualized energy requirements were estimated using the modified Harris–Benedict resting metabolic rate equation, revised by Mifflin et al.^18^ This was subsequently multiplied by a physical activity level (PAL) of 1.4 for the run‐in phase and 1.2 for the HDT phase. A standard diet was provided across all phases, with meals being the same for all participants but individualized for required energy intake by adjusting the amount of component foods given. Participants received three principal meals (breakfast, lunch and dinner) and one snack per day. Macronutrient composition of the diet was designed to provide 50–60% carbohydrates, ~30% fat and ~15% protein, and participants were encouraged to eat all food provided. Any food not eaten was weighed, and actual nutritional intake was recorded and subsequently analysed. See *Figure* *S1a* for schematics of the study design and *Figure* *S1b* for the experimental visit plan.

#### Experimental visits

Experimental visits were carried out on Day −4 (before BR), Day 3 (after BR) and Day 7 (after remobilization) of the protocol. On the day of each experimental visit (*Figure* *S1a*), participants received a standardized meal the evening before and then fasted from midnight. On the morning of each experimental visit, with participants in the fasted state, a muscle biopsy sample was obtained from the *vastus lateralis* with a second biopsy taken at 180 min of the hyperinsulinaemic–euglycaemic clamp (insulin infusion was maintained until after the biopsy was taken). Muscle biopsies were obtained using a 5‐mm Bergström needle, following local anaesthesia.^19^ Two passes through the same entry point were made on each occasion. Muscle biopsies of both legs were obtained on Day 7 for the calculation of MPS rates in the resistance exercise‐supplemented remobilized leg and contralateral non‐exercised leg. Additional muscle biopsies of the *vastus lateralis* were performed on Day 0 and Day 1 for the measurement of MPS during BR using a 12‐g (10‐cm) automated disposable Bard® Monopty® microbiopsy (Bard Ltd, Crawley, UK) needle. Muscle samples were immediately snap‐frozen in liquid nitrogen and stored at −80°C until analysed.

Cannulae were inserted retrograde into a superficial vein on the dorsal surface of the non‐dominant hand and anterograde into one arm at the antecubital fossa. The cannulated hand was kept in a hand‐warming unit (air temperature 55°C) to arterialize the venous drainage of the hand, and a slow 0.9% saline drip was attached to keep the cannula patent for repeated blood sampling. Following this, an anterograde femoral venous catheter was inserted (using the Seldinger technique under ultrasound guidance), to enable venous blood draining from the leg to be analysed for glucose concentration. Femoral venous catheterization was performed in the horizontal, supine position to reduce the risk of air embolism. A blood sample (1 mL) was taken from the dorsal heated hand cannula and the femoral venous line at baseline and at 120, 135, 150, 165 and 180 min of the insulin clamp to calculate arterialized‐venous versus venous (AV‐V) difference. Femoral artery blood flow in the contralateral limb was obtained at these same time points using ultrasonography to facilitate the calculation of leg glucose uptake.

An hyperinsulinaemic‐euglycaemic clamp was performed, with insulin (human Actrapid, Novo Nordisk) infused into the non‐dominant arm at a rate of 60 mU/m^2^/min for 3 h. Arterialized‐venous blood glucose concentration was measured in <1‐mL blood every 5 min and maintained at 4.5 mmol/L by a variable rate infusion of 20% w/v glucose (Baxter Healthcare, UK). Arterialized‐venous blood samples (3 mL) were taken at baseline and every 30 min during the final 60 min of the clamp for the measurement of insulin concentration. Once the final biopsy was completed, the insulin infusion was stopped, and participants were provided with a high‐carbohydrate meal. The glucose infusion was gradually reduced as glucose from the gut appeared in the blood. Once the participants' blood glucose concentration had been stable for 30 min without the infusion, all lines were removed. In total, 149 mL of whole blood was collected over the course of the 11 days of the study protocol, which is highly unlikely to have impacted on the experimental endpoint measures made. During the experimental visit after remobilization (Day 7), an additional pre‐clamp muscle biopsy was performed on the non‐exercised (ambulation only) leg using a microbiopsy needle for quantification of MPS to allow comparison with the resistance exercise‐supplemented remobilized leg. Femoral venous cannulation was performed on the resistance exercise‐supplemented remobilized leg rather than the non‐exercised leg to determine the effect of resistance exercise on leg glucose uptake. The morning after each experimental visit, a 3‐T magnetic resonance imaging (MRI) scan was performed at the Sir Peter Mansfield Imaging Centre at the University of Nottingham to quantify whole‐body muscle and leg muscle volumes.

#### Ethical approval

All participants gave their informed written consent to participate in this study. The study was granted ethical approval by the University of Nottingham Medical School Ethics Committee (Ethics Reference 6‐1704). The protocol was registered at www.clinicaltrials.gov (references: NCT03495128 and NCT03594799).

### Analytical method details

#### Femoral artery blood flow, arterialized‐venous versus venous difference and leg glucose uptake

The femoral artery was located at the level of the inguinal crease (below the inguinal ligament) using Toshiba Diagnostic Ultrasound System (Model SSA‐77OA) and a 12‐ to 14‐MHz linear probe. Vessel diameter was measured using B‐mode imaging synchronized to a 3‐lead electrocardiogram (ECG), and blood flow velocity was determined using Doppler (as previously described^20^). Measurements were made on the contralateral leg to the femoral venous catheter due to technical difficulties of scanning above a cannula insertion site and to reduce the impact of non‐linear flow (as a result of the 0.9% saline infusion) confounding velocity measures. Further details are provided in the supporting information.

#### Blood analyses

Whole blood collected during the insulin clamp was immediately analysed for glucose concentration (YSI2300; Yellow Springs Inc., OH, USA). Serum insulin concentration was measured using a solid‐phase ^125^I human‐specific radioimmunoassay (Merck Millipore, Billerica, MA, USA).

#### Muscle glycogen content

An aliquot of snap‐frozen wet muscle tissue was freeze‐dried, visible blood and connective tissue were removed and the muscle was powdered. An aliquot of freeze‐dried muscle powder was extracted using 0.1‐mol/L NaOH for determination of glycogen content.^21^

#### Body water D_2_O enrichment

Body water and muscle protein D_2_O enrichment were measured as previously described.^22^ Further details are provided in the supporting information.

#### Calculation of myofibrillar protein fractional synthetic rate

The fractional synthetic rate (FSR) of myofibrillar proteins was determined using a D_2_O tracer as described by Wilkinson et al.^23^ Further details are provided in the supporting information.

#### Whole‐body muscle protein breakdown

A high‐performance liquid chromatography (HPLC; Dionex Ultimate 3000, Thermo Scientific)–mass spectrometric (MS; Q‐Exactive, Thermo Scientific) approach was used to estimate the rate of MPB, using the D_3_–3‐MeH enrichment decay over time,^24^ in response to BR (time points were Day 0 and Day 2), using the same method as described by Gharahdaghi et al.^25^ Enrichment decay (*k*) of 3‐MeH over the 7 h of sampling in plasma was calculated within the first 48 h of BR (from Day 0 to Day 2).

#### Muscle volume quantification using magnetic resonance imaging

MRI was performed using a Philips 3‐T Ingenia scanner (Philips, Best, The Netherlands) to quantify whole‐body muscle and leg muscle (all muscle of the gluteal muscles, quadriceps, adductors and hamstrings) volumes. Further details are provided in the supporting information.

#### Total ribonucleic acid extraction and targeted muscle messenger ribonucleic acid measurements

Total RNA was extracted from frozen muscle biopsies as previously described.^26^ Further details of methodologies for targeted muscle mRNA expression measurements are provided in the supporting information.

### Quantification and statistical analysis

All data were coded and analysed using SPSS Version 24.0 (Statistical Package for Social Sciences, Chicago, IL, USA) or GraphPad Prism Version 7 (GraphPad Software Inc., USA). Data were initially checked for normality of distribution (using the Shapiro–Wilk test). One‐way repeated‐measures analysis of variance (ANOVA) was performed to detect any main effects of visit (before BR, after BR and after remobilization) for LGU and whole‐body muscle volume and CSA. Two‐way repeated‐measures ANOVA was performed to detect any effect of visit (before BR, after BR and after remobilization) and leg (exercise remobilization and remobilization) on leg muscle volume. Post hoc analysis was performed using the Bonferroni post hoc test. Where appropriate, Student's paired *t* test (or its nonparametric equivalent for non‐normally distributed data) was employed to compare two points for repeated measures. All data are presented as mean ± SEM. Statistical significance was assumed where *P* < 0.05.

#### Sample size

The test–retest coefficient of variation for the hyperinsulinaemic‐euglycaemic clamp (separation duration 1 week) within our laboratory is 10%. Pilot studies by our group have demonstrated a 30% reduction in GD over 72 h of forearm immobilization. Thus, it was calculated that such an effect would be measurable in eight subjects with a power of 80% at a 5% significance level on a paired *t*‐test basis.

## Results

We recruited 10 healthy male participants (age 24 ± 1 years, body mass index [BMI] 22.7 ± 0.6 kg/m^2^, whole‐body mass 70.7 ± 3.2 kg, lean mass 56.6 ± 2.1 kg, fat mass 10.9 ± 3.8 kg and muscle volume 29 370 ± 1613 cm^3^) to undertake 3 days of −6° head‐down tilt BR, preceded by a 7‐day run‐in period and followed by 3 days of structured unilateral resistance exercise. The study was conducted at the University of Nottingham in accordance with the European Space Agency Standardization of Bed Rest Study Conditions guidelines.^27^ Throughout the study, participants were maintained in energy balance (energy intake and macronutrient composition presented in *Table* *S1*), and prior to BR intervention, participants had an average daily PAL of 1.50 ± 0.06, measured using an Actiheart, equating to between 9000 and 10 000 steps/day.^28^

### Acute bed‐rest reduced insulin‐stimulated leg glucose and muscle glycogen storage, which 3 days of exercise‐supplemented remobilization failed to restore

**Figure 1:**
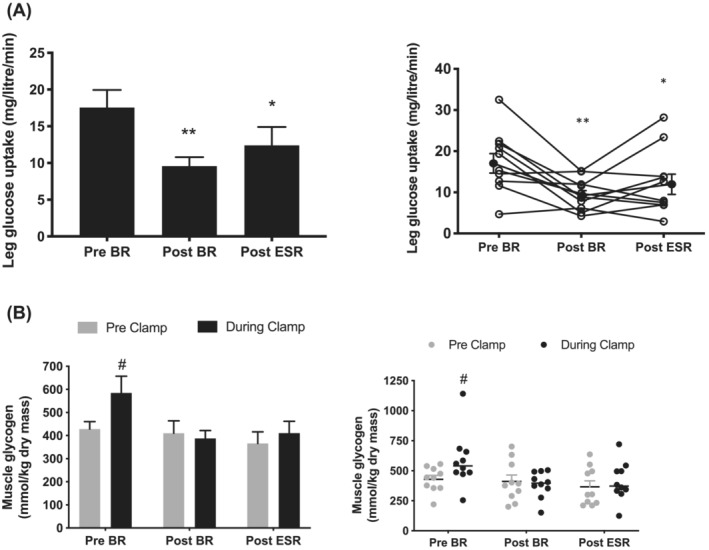
Acute bed‐rest reduced insulin‐stimulated leg glucose uptake and muscle glycogen storage, which exercise‐supplemented remobilization failed to restore. (A) Histogram depicting mean ± SEM steady‐state leg glucose uptake standardized to leg volume (litres) before bed‐rest (pre‐BR), after bed‐rest (post‐BR) and after exercise‐supplemented remobilization (post‐ESR) and corresponding individual responses (open circles) with mean ± SEM (closed circles). ^*^ *P* < 0.05, ^**^ *P* < 0.01 compared with corresponding pre‐bed‐rest value. (B) Muscle glycogen content (mean ± SEM) before (pre‐clamp) and during (during clamp) each insulin clamp, determined before bed‐rest (pre‐BR), after bed‐rest (post‐BR) and after exercise‐supplemented remobilization (post‐ESR). Corresponding individual values and mean ± SEM are also provided at all time points before clamp (grey circles) and during clamp (black circles). ^#^ *P* = 0.056 compared with pre‐clamp pre‐BR.

Compared with before BR, there was a 45% reduction in resting steady‐state LGU, standardized to leg muscle volume (measured using MRI), after BR (*P* < 0.01; *Figure* *1* *A*). In addition, LGU was not fully restored after 3 days of ESR of the limb, being 30% lower than before BR (*P* < 0.05). *Table* *1* summarizes the leg AV‐V difference and femoral arterial blood flow calculated at rest during the steady‐state of the insulin clamp at each time point (before BR, after BR and after ESR). The reduction in insulin‐stimulated LGU after BR was paralleled by a 38% reduction in leg blood flow compared with before BR (*P* < 0.01). Resting leg blood flow after the period of ESR was no different from before BR (*P* = 0.29). Compared with before BR, there were no differences in the steady‐state AV‐V balance during the clamp after BR (*P* = 1.00) or after remobilization (*P* = 0.99).

**Table 1:** Bed‐rest caused a decrease in insulin‐stimulated leg blood flow, with no effect on AV‐V difference, measured during the hyperinsulinaemic–euglycaemic clamp

	Time point	Steady‐state (average 135–165 min)
AV‐V difference (mmol/L)	Pre‐BR	1.44 ± 0.20
	Post‐BR	1.23 ± 0.11
	Post‐remobilization	1.24 ± 0.10
Leg blood flow (cm^3^/min)	Pre‐BR	424 ± 47
	Post‐BR	262 ± 24**
	Post‐remobilization	331 ± 36

*Note*: Steady‐state arterialized‐venous versus venous (AV‐V) difference (mmol/L) and femoral artery blood flow (cm^3^/min) measured before BR (pre‐BR), after BR (post‐BR) and after exercise‐supplemented remobilization (post‐remobilization). Values are mean ± SEM. Abbreviation: BR, bed‐rest.

** *P* < 0.01 compared with before BR by one‐way repeated‐measures analysis of variance with Bonferroni post hoc test.

*Figure* *1* *B* shows muscle glycogen content before (pre‐clamp) and in the insulin‐stimulated state (during clamp) at each time point (before BR, after BR and after ESR). Before BR, there was a 36% increase in muscle glycogen content as a result of the insulin clamp (*P* = 0.056). However, a corresponding increase in muscle glycogen content during the insulin clamp was not observed after BR (*P* = 0.69) or ESR (*P* = 0.57).

### Acute bed‐rest caused declines in myofibrillar protein synthesis and whole‐body muscle protein breakdown, with myofibrillar protein synthesis being restored by exercise‐supplemented remobilization but not ambulation alone

**Figure 2:**
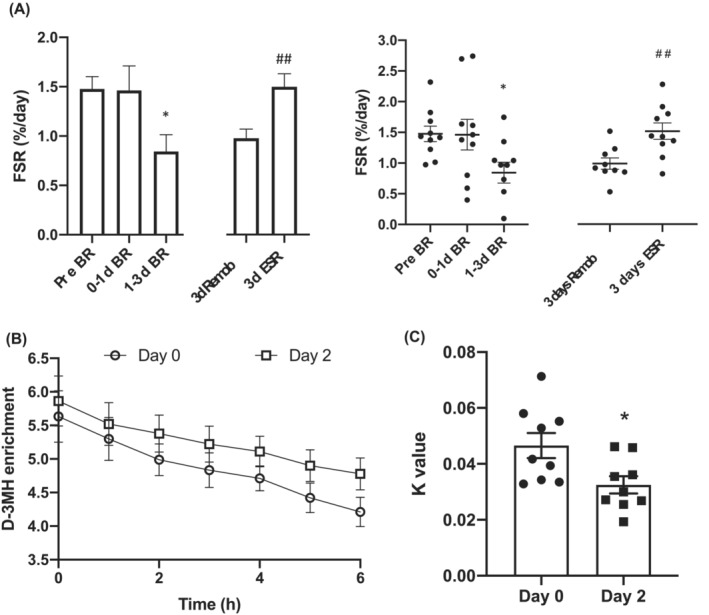
Acute bed‐rest caused declines in myofibrillar protein FSR and whole‐body MPB, and myofibrillar protein synthesis was restored by exercise‐supplemented remobilization but not ambulation alone. (A) Myofibrillar fractional synthetic rate (FSR, mean ± SEM) in %/day before bed‐rest (pre‐BR), after 24 h of bed‐rest (0‐1d BR), and between Days 1 and 3 of bed‐rest (1‐3d BR) *P < 0.05 versus pre‐BR (Student's *t* test) and FSR after remobilization in the exercised leg (3 days exercise‐supplemented remobilization [ESR]) and the non‐exercised leg (3 days Remob). ^##^ *P* < 0.1 versus non‐exercise leg (Student's *t* test). Corresponding individual values and mean ± SEM are also provided at all time points. (B) Isotopic enrichment of 3‐MeH in plasma the day following oral stable isotope tracer administration on Day 0 and Day 2 of bed‐rest. (C) Corresponding enrichment decay constants (*k*), mean ± SEM and individual values. *P < 0.05 versus Day 0 (Student's *t* test).

Compared with before BR, myofibrillar protein FSR was unchanged over the first day of BR but had declined by 43% by Day 3 (*P* < 0.05, *Figure* *2* *A*). Myofibrillar protein FSR was fully restored to pre‐BR rates by ESR, but FSR in the non‐exercised leg was 35% lower than in the exercised leg (*P* < 0.01; *Figure* *2* *A*). Isotopic enrichment of D_3_–3‐MeH in plasma on Day 0 and Day 2 is shown in *Figure* *2* *B*, and from this enrichment, decay rate constants (*k*) were calculated (*Figure* *2* *C*). Over the 7 h of sampling, the rate constant for plasma D_3_–3‐MeH dilution was reduced by 30% within the first 48 h of BR (from Day 0 to Day 2), indicating a reduction in whole‐body myofibrillar protein breakdown (*P* < 0.05).

### Acute bed‐rest caused a decrease in leg, but not whole‐body, muscle volume, which was restored by exercise‐supplemented remobilization but not ambulation alone

Neither absolute body mass nor whole‐body muscle volume (MRI‐derived) was different from before BR (70.7 ± 3.2 kg, 29 370 ± 1613 cm^3^) following BR (70.6 ± 3.2 kg, 29 116 ± 1616 cm^3^). *Table* *2* summarizes the percentage change in muscle volume for the whole body and legs. Compared with before BR, there was no change in whole‐body muscle volume after BR (*P* = 0.12) or after ESR (*P* = 0.64). However, BR resulted in a −2.6% (*P* < 0.01) and −2.5% (*P* < 0.01) reduction in the right and left leg muscle volume (MRI‐derived), respectively, which was restored in the exercise‐supplemented remobilized leg but not the leg exposed to ambulation alone (*P* < 0.01; *Table* *2*).

**Table 2:** Acute bed‐rest caused a decrease in leg, but not whole‐body, muscle volume, which was restored by exercise‐supplemented remobilization but not ambulation alone

A. Whole‐body muscle volume % change			
	Time point	% change	
Whole‐body muscle volume	Post‐bed‐rest	−0.7 ± 0.3%	
	Post‐remobilization	−0.5 ± 0.3%	

B. Leg muscle volume % change in the exercise‐supplemented leg (exercise + remobilization) and the non‐exercise‐supplemented leg (remobilization), measured using MRI			
	Time point	Exercise + remobilization	Remobilization
Leg muscle volume % change	Post‐bed‐rest	−2.6 ± 0.5%**	−2.5 ± 0.6%**
	Post‐remobilization	−0.7 ± 0.3%	−2.3 ± 0.3%**

*Note*: Values are mean ± SEM. Abbreviation: MRI, magnetic resonance imaging.

** *P* < 0.01 compared with pre‐bed‐rest by two‐way repeated‐measures analysis of variance (visit and leg) with Bonferroni post hoc test.

### Muscle messenger ribonucleic acid changes occurred after bed‐rest and exercise‐supplemented remobilization but did not reflect the physiological and metabolic events observed

**Figure 3:**
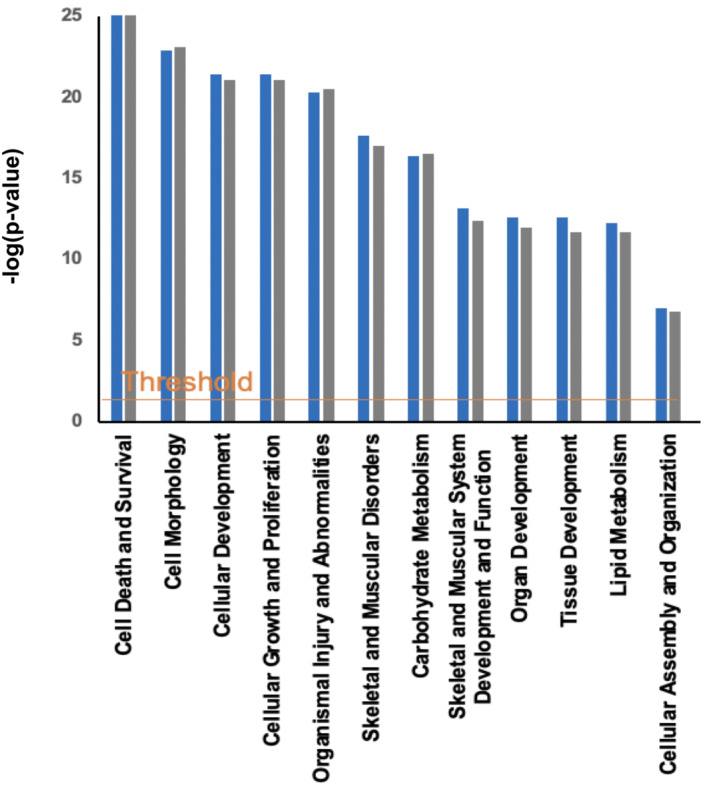
Muscle mRNA changes occurred after bed‐rest and exercise‐supplemented remobilization but did not reflect the physiological and metabolic events observed. Cellular functions identified by IPA as being most altered in muscle as a result of bed‐rest (blue) and exercise‐supplemented remobilization (grey) relative to before bed‐rest, based on mRNA expression data generated using the low‐density micro‐array cards. The *x* axis displays cellular functions most affected by bed‐rest, and the *y* axis displays the −log of the *P* value. The threshold line corresponds to a fold change of 1.5 and a *P* value of 0.05.

**Figure 4:**
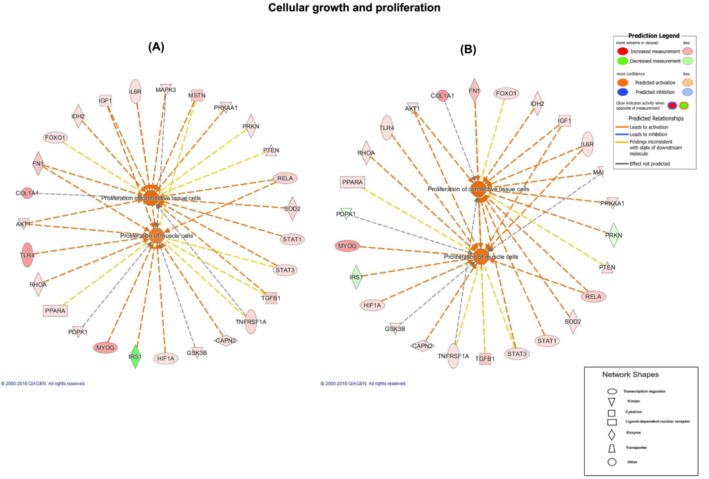
Pathway analysis for ‘cellular growth and proliferation’. Ingenuity pathway analysis schematic highlighting mRNAs differentially regulated from baseline in the ‘cellular growth and proliferation’ network (outer ring) and the predicted cellular events (inner octagons) associated with these collective changes (A) after bed‐rest and (B) after exercise‐supplemented remobilization compared with before bed‐rest.

Muscle mRNA expression of 191 target genes (*Table* *S2*) was quantified using low‐density array gene cards in the pre‐insulin clamp biopsies (fasted state) before and after BR and after the period of ESR. Ingenuity pathway analysis (IPA) identified 12 cellular functions as being significantly altered following BR, and structured remobilization, relative to before BR (*Figure* *3*). Notably, the magnitude of response [−log (*P* value)] was similar for each scenario. To illustrate this latter point, for the cellular function ‘cellular growth and proliferation’, 26 transcripts had increased in abundance and 1 declined after BR, with 23 transcripts being increased in abundance and 3 decreased after exercise remobilization (*Figure* *4*). Based on these collective responses, IPA predicted activation of ‘proliferation of connective cell tissues’ and ‘proliferation of muscle cells’ with high confidence for both experimental situations.

For the cellular function ‘carbohydrate metabolism’, 39 transcripts had increased in abundance after BR compared with before BR and 1 decreased in abundance (*Figure* *S2*). IPA strongly predicted the activation of ‘glycolysis’, ‘metabolism of carbohydrate’ and ‘synthesis of carbohydrate’ and ‘synthesis of glycogen’. After ESR, 29 transcripts were increased in abundance compared with before BR and 7 were decreased in abundance, with the activation of ‘quantity of carbohydrate’ and ‘synthesis of carbohydrate’ being predicted.

## Discussion

Here, we show that 3 days of BR reduced insulin‐stimulated LGU and muscle glycogen storage, with neither being restored to the pre‐BR state after 3 days of resistance ESR. Furthermore, this BR‐induced reduction in LGU was explained by a reduction in leg blood flow during the insulin clamp. BR also caused a reduction in leg muscle volume and the rate of MPS, alongside an adaptive decline in whole‐body MPB. However, resistance ESR, but not ambulation alone, was able to restore the decrease in leg muscle volume and rate of MPS. These findings provide novel insight of the regulation of LGU and muscle mass during immobilization and subsequent structured short‐term remobilization in humans. The findings also have important implications for ambulation and rehabilitation strategies in people following a period of BR.

A primary finding from this study was the failure of 3 days of resistance ESR to restore deficits in insulin‐stimulated LGU or muscle glycogen storage induced by BR. This decrement in LGU during the insulin clamp after BR appeared to be largely explained by a reduction in leg blood flow. Our findings confirm previous studies, which have demonstrated a reduction in limb glucose uptake after 24 h of unilateral forearm immobilization (which was isolated to the immobilized limb)^5^ and 7 days of BR.^4^ Previous studies have reported vascular dysfunction after BR, including a reduction in calf blood flow after 5 days of BR, measured using venous occlusion plethysmography.^29^ Other studies also report a diminished effect of insulin on the stimulation of skeletal muscle blood flow, both in the forearm (measured with venous occlusion plethysmography) after 10 days of BR^30^ and in the leg after 7 days of BR, with a preserved arterialized‐venous (AV) difference across the leg.^4^ Importantly, the deficits in LGU and glycogen storage in our study persisted after ESR. Evidence suggests that a single bout of resistance exercise improves insulin sensitivity in healthy volunteers, measured using an intravenous insulin tolerance test.^31^ However, it is unclear what type, intensity and duration of activity are required to fully restore muscle insulin sensitivity following BR or what duration of free living alone would completely restore it. In a previous 21‐day BR study in healthy, sedentary young males maintained in energy balance, 4 days of normal ambulation was unable to restore whole‐body glucose tolerance (measured using an OGTT), and 5–14 days of normal ambulation was required to restore impaired glucose tolerance.^8^ We are not aware of any study that has examined the effect of ESR on both insulin‐stimulated LGU and muscle glycogen storage following short‐duration disuse, and the present findings show that BR‐induced impairement of LGU and storage persists under these conditions. It would be of interest to determine if more prolonged submaximal exercise during remobilization, rather than resistance exercise, could restore these deficits.

Alongside the reductions in LGU and storage after BR, there was a decrease in MPS, which coincided with a ~2.5% decrease in leg volume. However, there was also a decrease in whole‐body MPB over the initial 2 days of BR. Early studies utilizing intravenous amino acid tracer techniques to estimate acute (~3–4 h) MPS reported a decline in both fasted and fed MPS following >7 days of immobilization.^11^, ^12^ However, these studies do not inform upon more chronic MPS responses over time and studies evaluating chronic MPS rates in short‐duration immobilization are lacking. A study utilizing 4 days of unilateral leg immobilization in healthy males demonstrated reductions in chronic MPS with no change in MPB reported.^1^ We observed a decline in MPS between 1 and 3 days of BR but not within the first 24 h of the onset of immobilization. A previous study reported no change in MPS after 2 days of unilateral lower limb suspension,^32^ but to our knowledge, we report the earliest changes in integrated MPS thus far. The discrepancy in our MPB findings with those of Brook et al. may be due to the different models of immobilization employed, the different approaches in measuring MPB, the time frame during which MPB was determined (2 vs. 4 days) and/or that volunteers in the current study were maintained in energy balance, and therefore, daily energy intake was reduced during BR, which reduced the rate of MPB. Tesch et al. reported an increase in MPB after 72 h of unilateral leg immobilization, demonstrated by a 44% increase in interstitial 3‐methylhistidine within the *vastus lateralis* measured using microdialysis.^15^ However, the authors in this paper did not measure local blood flow, so the elevated 3‐MeH could have been attributed to decreased washout from the tissue as a result of immobilization.^33^ In longer duration immobilization, a study estimating MPB during a 9.5‐ and 15‐day duration spaceflight, and a 17‐day BR experiment, showed no change in urinary 3‐MeH excretion in either spaceflight or BR.^34^ A further study utilizing deuterated water determined MPB to be lower in older individuals during 2 weeks of lower limb immobilization compared with a 2‐week retraining period.^35^ Whilst we found that whole‐body MPB was in fact decreased within the first 2 days of BR, our data do not definitively inform on what was happening beyond 2 days of BR or what was occurring at a muscle cellular level.

Three days of resistance ESR, but not ambulation alone, was able to restore BR‐associated decreases in MPS. This was paralleled by the restoration of leg muscle volume in the same limb. Studies examining the restoration of MPS *in vivo* in humans following a short period (<5 days) of remobilization after BR are lacking. Rodent studies have found MPS rates to be restored in as little as 6 h after 7 days of hindlimb immobilization of the *gastrocnemius*.^36^ However, the metabolic rates of rodents are substantially higher compared with humans, limiting the generalization of these findings. Previous studies in humans have shown restoration of MPS in the leg following remobilization after 5 days of BR. However, these have been after prolonged high‐intensity resistance exercise rehabilitation programmes of 8‐week duration.^9^ The design of this study allows for the comparison with the contralateral non‐remobilized leg to scrutinize the impact of resistance exercise plus normal ambulation, versus normal ambulation alone, on the restoration of MPS. These findings have important clinical implications as they indicate that MPS is acutely sensitive to a resumption of muscular contraction via a short period of resistance exercise of an intensity known to exert an anabolic stimulus, but a more modest resumption of muscle contraction (in the form of ambulation) is insufficient to fully restore MPS rates and muscle mass to baseline after only 3 days. This should be considered when designing countermeasures following short periods of disuse, such as following acute illness or a hospital stay, which is currently not the case in UK clinical care packages.

Based on muscle mRNA expression changes from before BR, IPA identified 12 cellular functions that were significantly altered following BR and structured remobilization and notably that the magnitude of response [−log (*P* value)] was similar for each scenario. Similarly, the cellular events predicted by IPA to arise from these changes in cellular functions were similar following BR and resistance ESR. For example, in the case of ‘cellular growth and proliferation’, where one might reasonably expect differences in the direction of change of mRNAs between the BR state and structured remobilization, IPA predicted changes in cellular events with high confidence that were very similar, namely, ‘proliferation of connective tissue cells’ and ‘proliferation of muscle cells’, despite measurable differences in MPS and leg muscle volume after BR and resistance ESR. We reported muscle mRNA changes previously in genes related to carbohydrate and lipid metabolism^2^ in acute BR, where we saw extensive changes in muscle mRNA networks associated with muscle fuel metabolism, but these did not predict whole‐body physiological changes observed in whole‐body glucose uptake and fuel oxidation after acute BR. In comparison, after 56 days of BR, the muscle gene networks identified by IPA were far smaller, but they did predict the BR‐induced change in fuel oxidation at 56 days. Collectively, these findings point to acute mRNA abundance changes as not being reflective of the regulation of muscle metabolism during acute BR and short‐term exercise rehabilitation following BR. In keeping with this, establishing temporal resolution from both a physiology perspective (i.e., quantifying muscle adaptation with exercise training and immobilization) and frequency of tissue sampling has been highlighted as being critical to better understanding of the regulation of muscle adaptation to stress.^37^ Human volunteer studies have usually quantified the magnitude of physiological adaptation to a given stressor (e.g., exercise training) and collected muscle tissue samples at pre‐intervention and post‐intervention time points (i.e., 2‐point investigation). However, such an approach will miss dynamic tissue‐level responses, which by their very nature will not reflect the prevailing physiological state at that time.

In summary, acute BR caused a decline in insulin‐stimulated LGU and muscle glycogen storage in healthy, young volunteers, as well as reductions in muscle volume, leg MPS and whole‐body MPB in the same individuals. However, 3 days of resistance ESR after BR restored muscle volume and MPS, but LGU and muscle glycogen storage remained depressed. Novel insight of the concurrent regulation of LGU and muscle mass during immobilization and subsequent short‐term structured remobilization in humans has been provided, which also has implications for maximizing effective recovery following short‐duration immobilization or BR.

## Conflict of interest statement

The authors declare that they have no conflicts of interest.

## Supporting information


**Table S1.** Energy intake in kilojoules (kJ/day) and macronutrient content (g/day) prescribed and actual, before, during and after bed‐rest. Values before and after bed‐rest were calculated with a physical activity level (PAL) of 1.4, whilst the bed‐rest phase was calculated with a physical activity level of 1.2. Data are mean ± SEM.
**Table S2.** List of genes selected for mRNA expression measurements using TaqMan low‐density array gene cards.
**Figure S1.** Bed‐rest schema and experimental visit plan. Schematic indicating a) Schedule of experimental sessions, MRI scans, muscle biopsies, tracer ingestion and remobilisation. D_2_O, deuterium oxide, 3MeH, 3‐methylhistidine b) experimental session schema. I.V., intravenous. Non‐bold arrows indicate a microbiopsy was performed.
**Figure S2.** Ingenuity Pathway Analysis schematic highlighting mRNAs differentially regulated from baseline in the carbohydrate metabolism network (outer ring) and the predicted cellular events (inner octagons) associated with these collective changes a) after bed‐rest and b) after remobilisation compared with pre bed‐rest.
