# Supplementary material for: Bed‐rest and exercise remobilization: Concurrent adaptations in muscle glucose and protein metabolism
Source: J Cachexia Sarcopenia Muscle. 2024 Feb 12;15(2):603–14. doi: 10.1002/jcsm.13431 (PMC10995277; doi:10.1002/jcsm.13431)
Supplement: Supplementary file 1 — Table S1. Energy intake in kilojoules (kJ/day) and macronutrient content (g/day) prescribed and actual, before, during and after bed‐rest. Values before and after bed‐rest were calculated with a physical activity level (PAL) of 1.4, whilst the bed‐rest phase was calculated with a physical activity level of 1.2. Data are mean ± SEM. Table S2. List of genes selected for mRNA expression measurements using TaqMan low‐density array gene cards. Figure S1. Bed‐rest schema and experimental visit plan. Schematic indicating a) Schedule of experimental sessions, MRI scans, muscle biopsies, tracer ingestion and remobilisation. D2O, deuterium oxide, 3MeH, 3‐methylhistidine b) experimental session schema. I.V., intravenous. Non‐bold arrows indicate a microbiopsy was performed. Figure S2. Ingenuity Pathway Analysis schematic highlighting mRNAs differentially regulated from baseline in the carbohydrate metabolism network (outer ring) and the predicted cellular events (inner octagons) associated with these collective changes a) after bed‐rest and b) after remobilisation compared with pre bed‐rest. [file JCSM-15-603-s001.docx]

**Supporting Information**

**Methods**

**Study run-in phase, bed-rest phase and remobilization phase**

*Run-in phase*

The habitual activity of participants was objectively measured during the run-in phase over a period of 7 consecutive days using a triaxial accelerometer (Actiheart™, CamNtech Ltd, UK). Subsequently, participants were asked to maintain their normal levels of daily activity but not to engage in formal exercise 3-days prior to the first hyperinsulinaemic euglycaemic clamp (experimental visit 1). Participants received the oral stable isotope tracer deuterium oxide (D_2_O/ Heavy water) to quantify cumulative MPS rates. An initial oral bolus of 3mg/kg D_2_O was administered at 09:00am (divided into 3 equal doses 30 minutes apart to reduce the risk of side effects) on day -4 prior to bedrest, with individualised top-ups on day 0 and day 3 being given; calculated based upon the rate of decay of water and total body water of 60% in men. For example, in a 75kg man, the initial loading bolus was 225ml followed by a top up of 37ml on day 0 and 68 ml on day 3. This was with the aim to label and maintain the body water pool to ~0.2% atom percent excess (APE). To monitor the body water enrichment throughout the study, saliva samples (1ml) were collected prior to and 2 hours after each D_2_O ingestion, as well as additional saliva samples at 09:00 on day 0, day 1 and day 7 of the protocol. They were advised not to eat or drink fluids 30 minutes prior to each saliva sample. Samples were cold centrifuged at 3,000g to remove any debris that may have been present and then aliquoted into 2-ml vials and frozen at -80^o^C until analysis.

### Bed-rest phase

Participants underwent 3 days and 4 nights of -6^o^ head down tilt bed-rest in the University of Nottingham, UK. Details of the protocol have been described previously [2]. During the bed-rest phase, the stable isotope tracer D_3_-3-methylhistidine (D_3_-3-MeH) was used to determine the rate of whole-body myofibrillar protein breakdown. Ten mg was ingested (dissolved in 50ml distilled water) on day -1 prior to the bed-rest period and day 1 of bed-rest at 09:00am. For the collection of plasma of D_3_-3-MeH plasma enrichment, an anterograde venous cannula was inserted into the arm at the antecubital fossa 24-hours later (day 0 and day 2 of bed-rest) for sampling of venous blood every hour for a total of 7-hours. On the morning of day 3 (i.e. after 72-hours of bed-rest), after an overnight fast, participants underwent the second experimental visit. After this, a gradual, supervised return to ‘upright’ in the bed was carried out, with continuous cardiovascular monitoring to avoid postural hypotension. Participants remained in bed but were allowed to sit in the upright position until the morning of day 4 (after 96-hours of bed-rest).

### Remobilisation phase

On the morning of day 4, after an overnight fast, participants were transferred (without weight-bearing or standing) to a wheelchair, then transported by taxi to the 3T MR scanner at the Sir Peter Mansfield Magnetic Resonance Centre for their post bed-rest MRI scans. Following this, participants were fed and allowed to return to standing in a controlled environment. They then underwent supervised rehabilitation at the David Greenfield Human Physiology Unit (DGHPU) comprising of 5 sets of 30 repetitions **(with a rest of 2 minutes between sets)** of maximal isokinetic knee extensions (90^o^ per second) on an isokinetic dynamometer (Cybex, Humac Norm) of their dominant leg. **Participants were seated in an upright position with back support and the hip and knee joint flexed at 90^o^. This protocol was specifically chosen in order to scrutinise the differential impact of a return to ambulation versus ambulation plus a structured resistance exercise programme on MPS, and** this protocol has **previously** been shown to provide an anabolic stimulus **that results in muscle hypertrophy** [16]. **Participants were** then allowed to return home. They returned on days 5 and 6 (days 2 and 3 of the remobilisation period) to undergo repeat supervised rehabilitation of their dominant leg as described above. At 08:00am on the morning of day 7, they presented to the DGHPU to undergo their final experimental visit. For safety monitoring, a D-dimer blood test was taken to rule out a deep vein thrombosis (DVT) formation during bed-rest. The participants completed the study after a final MRI scan was performed on day 8.

**Femoral artery blood flow, AV-V difference and leg glucose uptake**

Femoral artery blood flow was calculated from mean blood velocity (cm/sec) and femoral artery cross sectional area (cm^2^) x 60 to express flow as cm^3^/min.

Arterialised-venous to femoral venous (AV-FV) difference was calculated by subtraction of venous blood glucose concentration (mmol/ml) obtained from the femoral vein cannula, from the arterialised-venous blood glucose concentration (mmol/ml) obtained from the retrograde hand cannula in heated hand box.

Leg glucose uptake (mmol/min) was calculated as:

Leg glucose uptake (mmol/min) = AV-FV difference (mmol/ml) x blood flow (cm^3^/min). Leg glucose uptake was then standardised to leg volume (litres) and expressed as mg/litre/minute. Insulin-stimulated leg glucose uptake was defined as during the ‘steady state’ of the insulin clamp which was determined between min 135-165 of the clamp [S1].

**Body water D_2_O enrichment**

Pure fractions of body water were extracted by heating 100 μl of saliva in an inverted 2 ml autosampler vial for 4 h at 100°C. Vials were then placed upright on ice to condense extracted body water and transferred to a clean autosampler vial ready for injection. Body water (0.1 μl) was injected into a high‐temperature conversion elemental analyser (TCEA, Thermo Scientific, Hemel Hempstead, UK) connected to an isotope ratio mass spectrometer (Delta V Advantage, Thermo Scientific). For isolation of myofibrillar protein, 30–50 mg of muscle was homogenized in ice‐cold homogenization buffer [S2], rotated for 10 min, and the supernatant was collected after centrifugation at 13,000 *g* for 5 min at 4°C. The myofibrillar pellet was solubilized in 0.3 M NaOH and separated from the insoluble collagen by centrifugation, and the myofibrillar protein was precipitated using 1 M perchloric acid (PCA). Myofibrillar proteins were precipitated from the sample homogenate with 1 M PCA and separated by centrifugation. Protein‐bound amino acids were released using acid hydrolysis by incubating at 110°C in 0.1 M HCl in Dowex H^+^ resin slurry overnight before being eluted from the resin with 2 M NH_4_OH and evaporated to dryness; amino acids were then derivatized as their *n*‐methoxycarbonyl methyl esters (MCME). Dried samples were suspended in 60 μl distilled water and 32 μl methanol, and following vortex, 10 μl of pyridine and 8 μl of methylchloroformate were added. Samples were vortexed for 30 s and left to react at room temperature for 5 min. The newly formed *n*‐methoxycarbonyl methyl esters of amino acids were then extracted into 100 μl of chloroform. A molecular sieve was added to each sample for ∼20 s before being transferred to a clean glass Gas Chromatography insert, removing any remaining water by size exclusion absorption. Incorporation of deuterium into protein bound alanine was determined by gas chromatography–pyrolysis–isotope ratio mass spectrometry (GC-pyrolysis-IRMS, Thermo Scientific, Hemel Hempstead, UK) alongside a standard curve of known L‐alanine‐2,3,3,3‐d4 enrichment to validate measurement accuracy of the instrument.

**Calculation of muscle myofibrillar protein** **fractional synthetic rate (FSR)**

Myofibrillar protein FSR was ascertained using the precursor‐product approach, from the incorporation of deuterium labelled alanine into protein, using the enrichment of body water (corrected for the mean number of deuterium moieties incorporated per alanine, i.e., 3.7, and the total number of hydrogen within the MCME derivative, (11)) as the surrogate precursor labelling between subsequent biopsies. In brief, the standard equation is:

FSR (%.h-1) = [(δAla)]/[( δp) x t] x 100

where, δAla = deuterium enrichment (in delta per mil) of protein‐bound alanine between subsequent biopsies, δP = precursor enrichment (i.e. saliva D_2_O, corrected for 3.7 deuterium in Alanine, in delta per mil) and t, time between biopsies.

**Muscle volume quantification using MRI**

Participants lay supine head-first on the moveable MRI bed with their head resting on a pillow and their hands by their side. Their arms were secured into a fixed position using foam blocks to maintain the palms facing towards their body and a Velcro binder was placed across their body just proximal to their elbows to prevent their arms from falling outside the coil area. Two anterior dStream body coils and a posterior dStream bed coil (Philips, Best, Netherlands) were placed on the participants covering their clavicles to below their ankles. Imaging of the whole-body was carried out using a T2-TSE sequence acquired over 6 different slabs and merged together online using Philips scanner parameters. Each slab was acquired in a single breath-hold (particularly for the chest area), and consisted of a field of view of 448 x 560 x 300 mm, with a reconstruction voxel size of 1 x 1 x 1.5 mm. A total of 200 slices were acquired in 20 seconds, acquiring the whole-body images in around 6 minutes (taking into account bed motion and preparation steps for each slab). Water images were reconstructed online at the scanner using a Philips in-built product (mDIXON Quant package, Philips, Best, Netherlands) with scanner computer water images to measure muscle volume.

Whole-body and leg muscle volume were quantified manually using Horos™ DICOM medical imaging software Version 3 (GPL-3.0, Annapolis, MD USA). Whole-body MRI scans were acquired in the coronal (longitudinal) plane and slices were analysed in this orientation as the resolution was best in this plane. The whole-body image was analysed in individual anatomical regions which were defined as; left calf, right calf, left upper leg and gluteal muscles (leg), right upper leg and gluteal muscles (leg), torso, left arm and right arm. Intrinsic muscles of the hands and feet and muscles of the face were not quantified due to the small volumes and difficulty in delineating muscle from connective tissue and tendons. Anatomical boundaries were defined as follows;

Torso – All muscle between shoulder joint and sacroiliac joint including abdominals but excluding thoracic and abdominal viscera

Arm – All muscle between wrist joint and shoulder joint

Leg – All muscle of the gluteal muscles, quadriceps, adductors and hamstrings.

Calf – All muscle between ankle joint and knee joint

Whole-body – All of the above combined

Muscle CSA (cm^2^) was quantified every 10 slices. A region of interest (ROI) was drawn around the outer perimeter of the muscle. Where there were several large muscle groups making up the anatomical region, several ROIs were drawn and added together. The CSA was then calculated for the slices not manually measured for each anatomical region using the formula:

CSA a + CSA b / 2 *10

The area was then multiplied by 0.15 (slice thickness) to get volume in cm^3^.

**Targeted muscle mRNA expression measurements**

First strand cDNA was synthesised from 1 μg of total RNA using random primers (Promega, Southampton, UK) and Superscript III (Invitrogen Ltd, Paisley, UK). mRNA expression measurements (191 targets; Supplementary Table 4) were made in 100 ng of cDNA loaded per channel using the Applied Biosystems 384-well microfluidics TaqMan array cards (Thermo Fisher Scientific Ltd, Loughborough, UK) The mRNAs investigated were deemed to be representative of insulin sensitivity, carbohydrate and fat metabolism, inflammation, and protein turnover according to our own published research findings [S3-5] and a search of the literature, and SA Biosciences (<http://www.sabiosciences.com/>) and IPA databases (<https://analysis.ingenuity.com/>). Data were further analysed using Applied Biosystems RQ Manager software (Thermo Fisher Scientific Ltd, Loughborough, UK) where the threshold level was normalized across all plates before Ct values were calculated for each gene target and sample. Relative quantification of mRNAs of interest was measured using the 2^−ΔΔCt^ method with hydroxymethylbilane synthase (HMBS) as the endogenous control, with the mean of the baseline sample used as the calibrator. To associate a biological function to the identified probe sets, Ct values were uploaded to IPA software (Redwood City, CA, USA) for pathway analysis of gene expression data.

Supplemental references:

S1. DeFronzo, R.A., J.D. Tobin, and R. Andres. (1969). Glucose clamp technique: a method for quantifying insulin secretion and resistance. Am J Physiol, 237(3): p. E214-23.

S2. Wilkinson, D. J., M. V. Franchi, M. S. Brook, M. V. Narici, J. P. Williams, W. K. Mitchell, N. J. Szewczyk, P. L. Greenhaff, P. J. Atherton, and K. Smith. (2014). A validation of the application of D(2)O stable isotope tracer techniques for monitoring day-to-day changes in muscle protein subfraction synthesis in humans, Am J Physiol Endocrinol Metab, 306: E571-9.

S3. Murton, A.J., et al., (2014). Transient transcriptional events in human skeletal muscle at the outset of concentric resistance exercise training, in J Appl Physiol (1985*)*. p. 113-25.

S2. Porter, C., D. Constantin-Teodosiu, D. Constantin, B. Leighton, S. M. Poucher, and P. L. Greenhaff. (2017). Muscle carnitine availability plays a central role in regulating fuel metabolism in the rodent, J Physiol, 595: 5765-80.

S3. Stephens, F. B., Wall, B.T., Marimathu, K., Shannon, C.E., Constantin-Teodosiu, D., Macdonald, I.A., et al. Skeletal muscle carnitine loading increases energy expenditure, modulates fuel metabolism gene networks and prevents body fat accumulation in humans. *J Physiol* 591, 4655-4666 (2013).

**Supplementary Table 1.** Energy intake in kilojoules (kJ/day) and macronutrient content (g/day) prescribed and actual, before, during and after bed-rest. Values before and after bed-rest were calculated with a physical activity level (PAL) of 1.4, whilst the bed-rest phase was calculated with a physical activity level of 1.2. Data are mean ± SEM.

| **Study period** | **Parameter** | **Value** |
| --- | --- | --- |
| Run-in  (PAL 1.4) | Prescribed energy intake (kJ/day) | 11041 ± 338 |
|  | Actual energy intake (kJ/day) | 10960 ± 319 |
|  | Difference between prescribed vs actual (kJ/day) | -287 ± 88 |
|  | Carbohydrate (g/day) | 291 ± 14 |
|  | Protein (g/day) | 83 ± 4 |
|  | Fat (g/day) | 79 ± 4 |
| Bed-rest  (PAL 1.2) | Prescribed energy intake (kJ/day) | 8792 ± 275 |
|  | Actual energy intake (kJ/day) | 8950 ± 244 |
|  | Difference between prescribed vs actual (kJ/day) | 158 ± 23 |
|  | Carbohydrate (g/day) | 250 ± 26 |
|  | Protein (g/day) | 73 ± 8 |
|  | Fat (g/day) | 68 ± 5 |
| Remobilisation  (PAL 1.4) | Prescribed energy intake (kJ/day) | 10555 ± 301 |
|  | Actual energy intake (kJ/day) | 10285 ± 323 |
|  | Difference between prescribed vs actual (kJ/day) | -270 ± 78 |
|  | Carbohydrate (g/day) | 316 ± 34 |
|  | Protein (g/day) | 91 ± 9 |
|  | Fat (g/day) | 86 ± 10 |

**Supplementary Table 2.** List of genes selected for mRNA expression measurements using TaqMan low-density array gene cards.

| **Gene symbol** | **Function** |
| --- | --- |
| 18S | Eukaryotic 18S rRNA |
| ACO1 | aconitase 1 |
| ACTA1 | actin, alpha 1, skeletal muscle |
| ADIPOQ | adiponectin, C1Q and collagen domain containing |
| AKT1 | v-akt murine thymoma viral oncogene homolog 1 |
| ALDH2 | aldehyde dehydrogenase 2 family (mitochondrial) |
| AMPD1 | adenosine monophosphate deaminase 1 |
| ATF4 | activating transcription factor 4 |
| ATF6 | activating transcription factor 6 |
| ATG12 | autophagy related 12 |
| ATG9A | autophagy related 9A |
| ATP2A1 | ATPase sarcoplasmic/endoplasmic reticulum Ca2+ transporting 1 |
| ATP2A2 | ATPase sarcoplasmic/endoplasmic reticulum Ca2+ transporting 2 |
| ATP2B2 | ATPase plasma membrane Ca2+ transporting 2 |
| ATP2B4 | ATPase plasma membrane Ca2+ transporting 4 |
| ATP5B | ATP synthase, H+ transporting, mitochondrial F1 complex, beta polypeptide |
| ATP5I | ATP synthase, H+ transporting, mitochondrial Fo complex, subunit E |
| ATP6AP2 | ATPase H+ transporting accessory protein 2 |
| B2M | beta-2-microglobulin |
| BECN1 | beclin 1, autophagy related |
| BRD4 | bromodomain containing 4 |
| CALM1 | calmodulin 1 |
| CALR | calreticulin |
| CAMK2A | calcium/calmodulin dependent protein kinase II alpha |
| CAMK4 | calcium/calmodulin dependent protein kinase IV |
| CANX | calnexin |
| CAPN1 | calpain 1, (mu/I) large subunit |
| CAPN2 | calpain 2, (m/II) large subunit |
| CARM1 | coactivator associated arginine methyltransferase 1 |
| CAS3 | caspase 3 |
| CASP8 | caspase 8, apoptosis-related cysteine peptidase |
| CASQ1 | calsequestrin 1 |
| casq2 | calsequestrin 2 |
| CAT | catalase |
| CCL19 | chemokine (C-C motif) ligand 19 |
| COL1A1 | collagen, type I, alpha 1 |
| COL2A1 | collagen, type II, alpha 1 |
| COl4A1 | collagen type IV alpha 1 chain |
| COL6A3 | collagen type VI alpha 3 chain |
| COX5A | cytochrome c oxidase subunit 5A |
| COX5B | cytochrome c oxidase subunit Vb |
| COX6B1 | cytochrome c oxidase subunit 6B1 |
| CPT1A | carnitine palmitoyltransferase 1A (liver) |
| CPT1B;CHKB-CPT1B | carnitine palmitoyltransferase 1B (muscle),CHKB-CPT1B readthrough (NMD candidate) |
| CRTC1 | CREB regulated transcription coactivator 1 |
| CS | citrate synthase |
| CTSL | cathepsin L |
| CUL4A | cullin 4A |
| CYCS | cytochrome c, somatic |
| DLAT | dihydrolipoamide S-acetyltransferase |
| DLD | dihydrolipoamide dehydrogenase |
| EIF2S1 | eukaryotic translation initiation factor 2, subunit 1 alpha, 35kDa |
| EIF4B | eukaryotic translation initiation factor 4B |
| EIF4E | eukaryotic translation initiation factor 4E |
| EIF4EBP1 | eukaryotic translation initiation factor 4E binding protein 1 |
| eno3 | fibronectin 1 |
| ERK1 | mitogen-activated protein kinase 3 |
| FABP3 | fatty acid binding protein 3, muscle and heart (mammary-derived growth inhibitor) |
| FBXO32 | F-box protein 32 |
| FGF21 | fibroblast growth factor 21 |
| FOXO1 | forkhead box O1 |
| FOXO3B;FOXO3 | forkhead box O3B pseudogene,forkhead box O3 |
| FST | follistatin |
| GAPDH | glyceraldehyde-3-phosphate dehydrogenase |
| Glud1 | glutamate dehydrogenase 1 |
| GPD2 | glycerol-3-phosphate dehydrogenase 2 (mitochondrial) |
| GSK3B | glycogen synthase kinase 3 beta |
| HADH | hydroxyacyl-CoA dehydrogenase |
| HIF1A | hypoxia inducible factor 1, alpha subunit (basic helix-loop-helix transcription factor) |
| HK2 | hexokinase 2 |
| HMBS | hydroxymethylbilane synthase |
| HSPA8 | heat shock 70kDa protein 8 |
| IDH2 | isocitrate dehydrogenase (NADP(+)) 2, mitochondrial |
| IER2 | immediate early response 2 |
| IFI30 | IFI30, lysosomal thiol reductase |
| IGBP1 | immunoglobulin (CD79A) binding protein 1 |
| IGF1 | insulin-like growth factor 1 (somatomedin C) |
| IGFBP1 | insulin like growth factor binding protein 1 |
| IKBKB | inhibitor of kappa light polypeptide gene enhancer in B-cells, kinase beta |
| IL6 | interleukin 6 (interferon, beta 2) |
| IL6R | interleukin 6 receptor |
| INSR | insulin receptor |
| IRS1 | insulin receptor substrate 1 |
| ITPR1 | inositol 1,4,5-trisphosphate receptor type 1 |
| ITPR3 | inositol 1,4,5-trisphosphate receptor type 3 |
| JKAMP | JNK1/MAPK8-associated membrane protein |
| JPH1 | junctophilin 1 |
| KCNT1 | potassium sodium-activated channel subfamily T member 1 |
| LDHA | lactate dehydrogenase A |
| M6PRBP1 | perilipin 3 |
| MAP1LC3A | microtubule-associated protein 1 light chain 3 alpha |
| MAPK9 | mitogen-activated protein kinase 9 |
| MCUR1 | mitochondrial calcium uniporter regulator 1 |
| MDH2 | malate dehydrogenase 2 |
| MNF1 | mitochondrial nucleoid factor 1 |
| MPC2 | mitochondrial pyruvate carrier 2 |
| MPST | mercaptopyruvate sulfurtransferase |
| MRF4 | myogenic factor 6 |
| MSTN | myostatin |
| MT1A | metallothionein 1A |
| MTFR1 | mitochondrial fission regulator 1 |
| MTOR | mechanistic target of rapamycin (serine/threonine kinase) |
| MYBPC1 | myosin binding protein C, slow type |
| Myf5 | myogenic factor 5 |
| MYH1 | myosin heavy chain 1 |
| MYH2 | myosin heavy chain 2 |
| MYH3 | myosin, heavy chain 3, skeletal muscle, embryonic |
| MYH4 | myosin, heavy chain 4, skeletal muscle |
| MYL1 | myosin, light chain 1, alkali; skeletal, fast |
| MYL6 | myosin, light chain 6, alkali, smooth muscle and non-muscle |
| MYLK2 | myosin light chain kinase 2 |
| MYLPF | myosin light chain, phosphorylatable, fast skeletal muscle |
| MYLPF | myosin light chain, phosphorylatable, fast skeletal muscle |
| MYO5C | myosin VC |
| MYOG | myogenin (myogenic factor 4) |
| NAT1 | N-acetyltransferase 1 |
| NAT2 | N-acetyltransferase 2 |
| NDUFB3 | NADH:ubiquinone oxidoreductase subunit B3 |
| NDUFB5 | NADH:ubiquinone oxidoreductase subunit B5 |
| NFE2L2 | nuclear factor, erythroid 2-like 2 |
| NFKB1 | nuclear factor of kappa light polypeptide gene enhancer in B-cells 1 |
| NRF1 | nuclear respiratory factor 1 |
| OGDH | oxoglutarate dehydrogenase |
| OPTN | optineurin |
| OSTN | osteocrin |
| OTOP1 | otopetrin 1 |
| PARKIN | parkin RBR E3 ubiquitin protein ligase |
| PAX3 | paired box 3 |
| PAX7 | paired box 7 |
| PDHA1 | pyruvate dehydrogenase (lipoamide) alpha 1 |
| PDIA2 | protein disulfide isomerase family A member 2 |
| PDK2 | pyruvate dehydrogenase kinase, isozyme 2 |
| PDK4 | pyruvate dehydrogenase kinase, isozyme 4 |
| PFKM | phosphofructokinase, muscle |
| PKM | pyruvate kinase, muscle |
| POLRMT | polymerase (RNA) mitochondrial (DNA directed) |
| PPARA | peroxisome proliferator-activated receptor alpha |
| PPARG | peroxisome proliferator-activated receptor gamma |
| PPARGC1A | peroxisome proliferator-activated receptor gamma, coactivator 1 alpha |
| PPP3CA | protein phosphatase 3 catalytic subunit alpha |
| PRKAA1 | protein kinase, AMP-activated, alpha 1 catalytic subunit |
| PRKAA2 | protein kinase, AMP-activated, alpha 2 catalytic subunit |
| PRKAB1 | protein kinase AMP-activated non-catalytic subunit beta 1 |
| PSMA1 | proteasome (prosome, macropain) subunit, alpha type, 1 |
| PSMC1 | proteasome (prosome, macropain) 26S subunit, ATPase, 1 |
| PSMC4 | proteasome 26S subunit, ATPase 4 |
| PTEN | phosphatase and tensin homolog |
| PYGM | phosphorylase, glycogen, muscle |
| RELA | RELA proto-oncogene, NF-kB subunit |
| RHOA | ras homolog family member A |
| RICTOR | RPTOR independent companion of MTOR, complex 2 |
| RPS6KB1 | ribosomal protein S6 kinase B1 |
| RPS6KB2 | ribosomal protein S6 kinase, 70kDa, polypeptide 2 |
| RPTOR | regulatory associated protein of MTOR, complex 1 |
| RYR1 | ryanodine receptor 1 |
| SDHA | succinate dehydrogenase complex, subunit A, flavoprotein (Fp) |
| SIX1 | SIX homeobox 1 |
| SLC39A6 | solute carrier family 39 member 6 |
| SMAD | SMAD family member 2 |
| SOCS3 | suppressor of cytokine signaling 3 |
| SOD2 | superoxide dismutase 2, mitochondrial |
| SREBF1 | sterol regulatory element binding transcription factor 1 |
| SREBP | sterol regulatory element binding transcription factor 1 |
| SRF | serum response factor |
| STAT1 | signal transducer and activator of transcription 1, 91kDa |
| STAT3 | signal transducer and activator of transcription 3 (acute-phase response factor) |
| STIM1 | stromal interaction molecule 1 |
| TFAM | transcription factor A, mitochondrial |
| TFB2M | transcription factor B2, mitochondrial |
| TGFB1 | transforming growth factor, beta 1 |
| TJP2 | tight junction protein 2 |
| TLR4 | toll-like receptor 4 |
| TNC | tenascin C |
| TNFR1 | TNF receptor superfamily member 1A |
| TNNC2 | troponin C2, fast skeletal type |
| TNNT1 | troponin T1, slow skeletal type |
| TRAF3 | TNF receptor-associated factor 3 |
| TRIM63 | tripartite motif containing 63, E3 ubiquitin protein ligase |
| TRPM1 | transient receptor potential cation channel subfamily M member 1 |
| TRPM7 | transient receptor potential cation channel subfamily M member 7 |
| TTN | titin |
| TUFM | Tu translation elongation factor, mitochondrial |
| UCP1 | uncoupling protein 1 (mitochondrial, proton carrier) |
| UCP3 | uncoupling protein 3 (mitochondrial, proton carrier) |
| YWHAZ | tyrosine 3-monooxygenase/tryptophan 5-monooxygenase activation |
| ZFAND5 | zinc finger AN1-type containing 5 |
| NDUFS6 | NADH:ubiquinone oxidoreductase subunit S6 |
| SCNN1A | sodium channel epithelial 1 alpha subunit |
| CTNNAL1 | alpha catenin |
| PDPK1 | 3-phosphoinositide-dependent protein kinase 1 |
| PIK3R5 | phosphoinositide-3-kinase regulatory subunit 5 |
| SLC2A4 | solute carrier family 2 member 4/Glut4 transporter |

**a**

**b**

**Supplementary Figure 1.** Bed-rest schema and experimental visit plan. Schematic indicating a) Schedule of experimental sessions, MRI scans, muscle biopsies, tracer ingestion and remobilisation. D_2_O, deuterium oxide, 3MeH, 3-methylhistidine b) experimental session schema. I.V., intravenous. Non-bold arrows indicate a microbiopsy was performed.

**b After remobilisation**


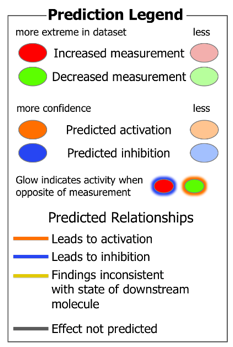


**Carbohydrate Metabolism**

**a) After bed-rest**


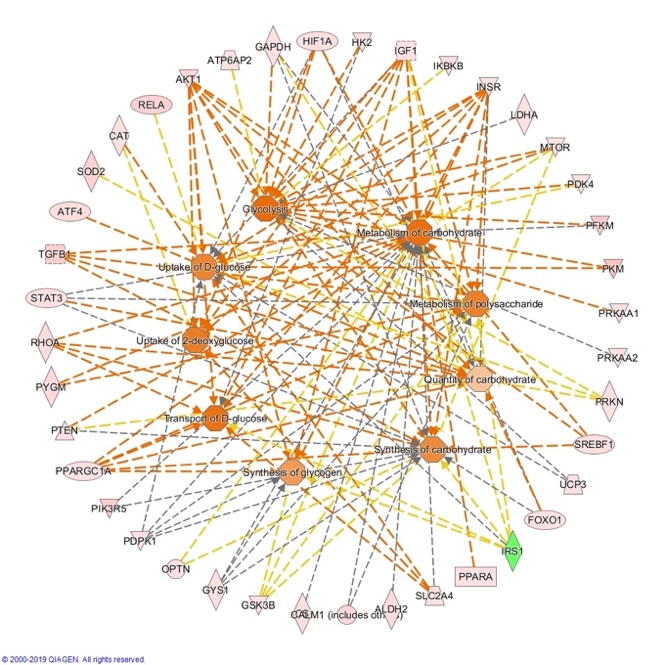

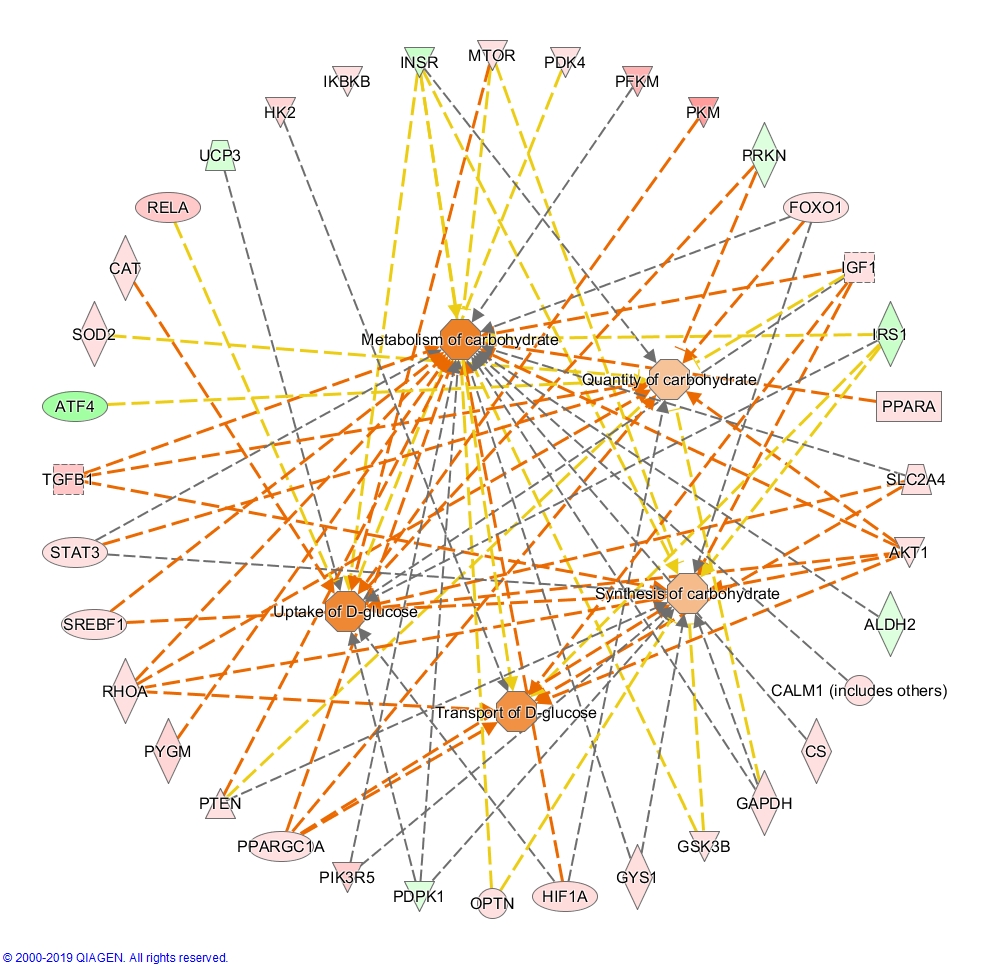


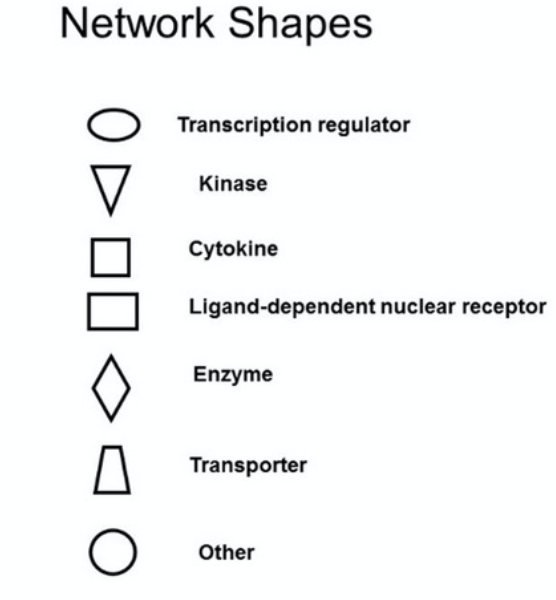


**Supplementary Figure 2**. Ingenuity Pathway Analysis schematic highlighting mRNAs differentially regulated from baseline in the carbohydrate metabolism network (outer ring) and the predicted cellular events (inner octagons) associated with these collective changes a) after bed-rest and b) after remobilisation compared with pre bed-rest.
